# US “Partnership” with the Egyptian Muslim Brotherhood and its Effect on Civil Society and Human Rights

**DOI:** 10.1007/s12115-013-9740-3

**Published:** 2013-12-18

**Authors:** Anne R. Pierce

**Affiliations:** Care of Department of Sociology (Attn: Jonathan Imber), Wellesley College, Pendleton East, 106 Central Street, Wellesley, MA 02481 USA

**Keywords:** American foreign policy, Human rights, Rule of law, Civil society, Barack Obama, Hillary Clinton, Arab Spring, Muslim Brotherhood, Christian Copts, Egypt, Mohamed Morsi, Abdel Fattah al-Sisi

## Abstract

Looking at Egypt before, during and after the Arab Spring, this paper examines the intersection of Christian Copts, the Muslim Brotherhood, the Egyptian army, moderate Muslims and secular groups. In turn, it examines the Obama administration’s policies toward Egypt. It discloses the surprising finding that the only *consistent* aspect of the administration’s policy toward Egypt has been outreach to and engagement with the Muslim Brotherhood. At no time before or after the Brotherhood’s ascent to prominence in Egyptian politics and society did the administration make support of the Brotherhood conditional. At no time did it use US leverage - given the massive amount of financial and military aid Egypt was depending on, and given the new Egyptian government’s desire for prestige in the world community–to pressure the Morsi government to respect human rights, religious liberty and the impartial rule of law. Arguing that American foreign policy at its best is rooted in democratic ideals, this paper asks whether the United States, while respecting that Egyptians must choose their leaders and their political system, could have done more to encourage a positive strategic, moral and political outcome.

American foreign policy at its best combines moral and practical concerns. It emphasizes the security of the free world (necessary in a world where global threats lie just beneath the surface) and the principles of freedom (essential to expanding the realm of human dignity and political liberty.) As the United States became a world power after World War II, it did so in terms that advanced both our defenses and our ideals. With the world reeling from the fascist assault, and facing the new threat of Soviet expansionism, America asserted influence as never before. While forming alliances and economic and strategic partnerships throughout the world, the United States also actively promoted democratic principles and sought to expand the number of representative democracies. Truman, Eisenhower, Kennedy and Reagan all insisted that our principles were what made our power important, and espoused those principles in their speeches and documents.

There were times when the United States sullied its own cause and credibility by making its enemy’s enemy a friend. But, the *overriding* goal during the postwar years was the expansion of the realm of political and economic freedom. Testimony to that emphasis: Most European and East Asian countries went from the postwar period of instability and hardship to democratic advances, economic prosperity, and relative external security. Europe and East Asia benefited from the American defense shield, from economic interaction with and aid from the United States, and from the projection of American/democratic ideals. When the Cold War ended with communist dictatorships collapsing one after the other, this was a victory not just for our military and material power, but also for the human rights and individual rights that the free world, at its best, embodies.

In spite of widespread assumptions to the contrary, even in the Middle East, post-World War II American policies included humanitarian and liberalization efforts. The United States pressured European powers to help prepare former enemy territories for “self-rule.” Roosevelt appointed Patrick J. Hurley to map out ways to seek “free governments and free enterprise,” and to put an end to “exploitation and imperialism.” Truman both decided to support the creation of a Jewish state in Israel and stated his belief that the peoples of the Middle East were “deserving of post-war political independence.” Eisenhower went so far as to support Egyptian President Gamal Abdel Nasser against the Europeans–even after Nasser shocked the world by announcing Egypt’s nationalization of the Suez Canal. When English and French planes bombed Egyptian airfields in response, the United States condemned the assault. But the United States would not be rewarded for supporting Egypt.

Increasingly under the sway of Soviet power and extremist ideals, many post World War II “self-determination” movements in the Middle East, including Nasser’s, started to define themselves less in terms of political freedom than in terms of Soviet-style “liberation” from capitalist, western powers, including the United States. By the time of Kennedy’s presidency, post-colonial regimes in Syria, Egypt, Iraq and elsewhere had devolved into dictatorships. As political and religious extremism rose, it was increasingly tempting to befriend authoritarian leaders who could help the United States hold extremists and/or communists at bay. Such was the case with the US alliance with Egyptian strongman Hosni Mubarak, a repressive leader whose transgressions the US mostly overlooked for the sake of Egypt’s cooperation on Israeli peace issues, terrorism and regional security.

Still, U.S. involvement in the Middle East continued to include financial aid, health, infrastructure and agriculture projects, and support of democratization. Some are so bold as to argue that, in spite of alleged American antipathy to Islam, most of America’s recent involvement in the Middle East has been intended to help Muslim people. Asserts Robert Lieber, “Remarkably, most of the post-Cold War American military interventions abroad have been to save Muslim populations from starvation, ethnic cleansing, civil war, invasion, and oppression–as large numbers of Kuwaitis, Somalis, Kurds, Bosnians, Kosovars, Iraqi Shiites, and the people of Afghanistan, especially women, can attest. Moreover, the absorptive character of the United States has made it far better than any of the countries of Europe or Asia in accommodating and integrating Muslims”^1^

By the time of the George W. Bush presidency, there were two currents shaking and transforming the Middle East–one toward Islamic extremism, the other toward political liberalization. Many in the west were blind to the later. They claimed that Arabs, being culturally different from the West, didn’t want democracy, and wouldn’t know what to do with it if they had it. They thought Middle Eastern nations were not “ready” for democracy because they had no democratic traditions. (Never mind that post-World War II Germany and Japan defied that logic; these Democratic allies had “traditions” that were far from democratic.) In addition, with cultural and moral relativism very much in vogue, influential intellectuals argued that anti-democratic regimes were just as valid as democratic ones, and that our best policy toward oppressive regimes was to refrain from judgment and find “common interests.”

The truth is that the majority of Middle Easterners longed for more freedom, both economically and politically. They were tired of the stagnation, corruption and repression of centralized regimes and sought alternatives to authoritarian governments. The Iraq War did not stem the tide of discontent; instead, democracy movements grew during the Bush years. The war did cause major resentment in the region, but so did realpolitik policies which had caused the United States to form reluctant alliances with Middle Eastern dictators. Indeed, claims that the United States supported governments that denied their people rights caused some middle easterners to turn to Islamic political movements as opposed to more secular/Western ones. Claims of U.S. collusion with repressive governments had been one of the selling points of Al Qaeda itself.

It was thus Western intellectuals, not Middle Easterners, who were upset by the Bush administration’s focus upon the Middle East’s “freedom deficit.” As the Arab Human Development Report and other internal surveys showed, sentiment against the freedom deficit was overwhelming in the Middle East itself by the time President Obama and Secretary Clinton took office. Unlike American intellectuals, Arab intellectuals who contributed to the post 9/11 Arab Human Development Report pushed the idea of democracy, stating, “The freedom deficit [in the Arab region] undermines human development and is one of the most painful manifestations of lagging political development.”^2^ Sentiment for more freedom was not limited to intellectuals. As Shibley Telhami shows in his analysis of public opinion polls, most people wanted human rights and democratization. Stated Telhami regarding the Arab Spring, “It was hardly surprising to discover Arabs were angry with their rulers. In fact, every year, after conducting the Annual Arab Public Opinion Poll in Egypt, Saudi Arabia, Morocco, Jordan, Lebanon, and the United Arab Emirates, the question that leapt from the findings was not, ‘When will Arabs have reason to revolt?’ but ‘Why haven’t Arabs revolted yet?”^3^

They *would* revolt soon thereafter, when the “Arab Spring” shook an entire region and opened up entirely new possibilities. What a pity that the Obama administration was completely unprepared for the situation and incapable of nurturing or influencing it. It is, of course, true that there was a parallel movement in the Arab world, for the Islamization of government, the imposition of Sharia Law and the conversion of infidels. With this the case, why wouldn’t the United States use its very substantial leverage and the steadfast enunciation of democratic principles to encourage the one and discourage the other? We should not attempt to dictate terms, but neither should we squander our influence or abet negative outcomes.

When Egyptians succeeded in overthrowing Hosni Mubarak, the time was right for the United States to reach out to the diverse peoples fighting for respect within Egyptian society, to help those people develop a framework for civil society and to form foreign policy toward Egypt that was grounded in both American interests and American principles. Instead, at no time did the Obama administration use US leverage-given the massive amount of financial and military aid Egypt was depending on, and given the new Egyptian government’s desire for prestige in the world community–to pressure the Egyptian government to respect individual rights, religious liberty and the impartial rule of law. Obama, Clinton and Panetta made the very occasional weak statement in favor of such things, but their policy was defined by *not* promoting these things; having at first defended the legitimacy of the Mubarak regime, they then defended the Muslim Brotherhood’s legitimacy, and abetted the group’s consolidation of power. Neither before nor after the Brotherhood’s ascent to prominence in Egyptian politics and society did the administration make that support conditional.

When we look at Egypt’s recent troubles we must ask whether the United States could have done more to encourage a positive strategic, political and moral outcome. It seems safe to say that clarity of vision and purpose on the part of the United States might have helped. As the revolution unfurled, an enthused U.S. stand for democracy and human rights were in order, as was an emphasis upon the benefits to the new Egyptian government of maintaining cooperative ties with the West and Israel, and supporting pluralism at home. Respect for sovereignty needn’t translate into doing nothing to foster political and religious freedom or to strengthen our strategic position. Precisely *because of* ascendant factions in Egypt desiring Islamic rule, a heartfelt stand for liberty as well as an attempt to maintain geopolitical influence were and are in order.

So, let’s retrace our steps, and look at missed opportunities and skewed priorities, not in order to place blame, but in order to move toward better policy. Egypt is a vibrant land and the revolt against Mubarak was filled with people from all walks of life and different religions who wanted more freedom, more opportunity, and less state interference in their lives. Their dreams have been thwarted by the Islamists in power and by the still-repressive military and security forces. But, as their protests in the streets indicate, their dreams have not died.

## Background to Revolution

Glenn Kessler of the *Washington Post* has documented the George W. Bush administration’s efforts (both behind the scenes and overt) to pressure the Mubarak government toward reform. In addition to criticizing the regime for its human rights abuses, Bush began to give money to democracy and good-governance programs and organizations that were independent of Mubarak’s regime. That effort, according to Kessler, was “largely shelved” by the Obama administration: “For fiscal year 2009, the administration immediately halved the money for democracy promotion in Egypt; the civil society funds were slashed 70 percent, to $7 million. Meanwhile, money that was to be given directly to civil society groups was eliminated and the administration agreed to once again fund only those institutions that had Mubarak’s seal of approval.”^4^ Funding for Voice of America-type programs in the Middle East was sliced as well.

Probably due to a combination of external and internal pressure, perhaps also reflecting differences with his father, by the time Obama came to power, Hosni Mubarak’s son Gamal was promising reforms that included privatizing Egypt’s economy and freeing it from the military’s grip. But, the long repressed Egyptian people didn’t trust Mubarak’s son, and didn’t want reform in the hands of Mubarak’s successor. They wanted free elections and the chance to choose their leaders. As Alaa al-Aswany explained in *On the State of Egypt*, “The argument that Gamal Mubarak will be a civilian president for Egypt is [also] based on a fallacy, because what defines the nature of a regime is not the profession of a president but the way in which he assumes power. … If Gamal Mubarak gains the presidency of Egypt, this will not put an end to military rule but merely add to it another disaster. Autocracy will be combined with a hereditary system, and after that what will there be to stop Gamal Mubarak from granting the presidency to his son or nephew?”^5^

Mubarak had stayed in power for thirty years through increasingly fraudulent elections. Asserts Shadi Hamid, “If there was any doubt the status quo was untenable, the November 2010 elections–arguably the most fraudulent in Egyptian history–confirmed what many long suspected: reform through the existing system had become impossible.”^6^ Corruption and cronyism were endemic to his regime, as were detentions by the dreaded security services. The regime had particularly targeted the Muslim Brotherhood who were subject to arbitrary arrests, torture and disappearances.

Interestingly, the Mubarak regime tolerated Egypt’s growing number of Islamic extremists, including Salafists, many of whom had been influenced by Wahhabism while working in Saudi Arabia or had been swayed by Wahhabi broadcasts and preachers. The reason, according to Al Aswani, is that Salafist Wahhabism actually *enables* despotic government as it urges Muslims to obey their rulers and forbids rebellion against Muslim leaders. Also interesting is the fact that, for the sake of appeasement and keeping Islamist violence at bay, Mubarak increasingly went along with Islamist demands to treat Christians like second-class citizens.^7^

Egypt’s Christian Copts are one of the most ancient Christian communities, tracing their roots back to the Gospel writer Mark who brought Christianity into Egypt in the first century. They are also the largest Christian community in the Middle East. As scholar Edward Wakin put it in *The Lonely Minority*, “The Copts are a major test of modern coexistence between a large Christian minority and a Muslim majority” and have been “the major transmitters of Western and modern attitudes in Egypt.”^8^ For most of the AD period, the Copts were a harshly persecuted minority. Their position did not begin to improve until the early 19th century, when Mohamed Said Pasha abolished the Jizya (a tax on non-Muslims) and allowed Copts to enroll in the army. Conditions continued to improve throughout the 19th century under the leadership of Pope Cyril IV, and in the first half of the 20th century (known as the *Golden Age* by the Copts). Copts participated in the Egyptian national movement for independence and occupied many influential positions.

In the second half of the twentieth century, from the time of Nasser and through the time of Sadat and Mubarak, the Copts faced discriminatory policies which generally fell short of outright persecution. The Copts were an important part of Egyptian society even though they lacked equal rights and equal protection under the law. Wakin documented the institutionalized stratification which favored Muslims, and the quotas for Christians in all areas of Egyptian life–government, education and business. Copts who dared to proselytize were regularly detained, while those who agreed not to make waves were granted certain benefits. Copts faced discrimination in matters such as housing and church construction permits, and faced major hurdles in vying for high positions in society and government. Those Muslims who chose to harass and intimidate Christians could generally do so with immunity.

Mubarak’s regime was hard for Christians, but it was not at all as hard as Iran’s or Somalia’s or North Korea’s or Saudi Arabia’s. And it was not nearly as hard as it would be under Mohammed Morsi. Coptic scholar Samuel Tadros explains, “Their country’s transformation wasn’t sudden, but every year brought more public Islamization. As the veil spread, Coptic women felt increasingly different, alien and marked. Verbal abuse came from schoolteachers, bystanders in the bus station who noticed the cross on the wrist, or commentators on state television. But life was generally bearable. He [Hosni Mubarak] was no friend to the Copts, but neither was he foe. His police often turned a blind eye when Coptic homes and shops were attacked by mobs, and the courts never punished perpetrators-but the president wasn’t an Islamist. He even interfered sometimes to give permission to build a church, or to make Christmas a national holiday.”^9^

Life was “generally bearable,” but the historic cycles of repression and the rise of Islamist extremism meant that there was reason for vigilance and the pronouncement and defense of democratic principles, not just on the part of Egyptian human and religious rights groups, but also on the part of those in positions of influence in the United States.

By the time President Obama came to office, oppression of Christians by Muslim extremists had intensified, and Mubarak’s government, under pressure itself, was doing little to stop it. In spite of Mubarak’s often successful attempts to convince the West that Egypt was pluralistic and tolerant, numerous sources indicated that Egyptian authorities were not offering effective protection to Copts who complained of harassment, attack or rape by Muslim perpetrators. Coptic Christian complainants were often pressured by government authorities to engage in “reconciliation sessions” and were sometimes arrested themselves as a way for the government to avoid prosecuting alleged Muslim assailants. In *Persecuted: The Global Assault on Christians,* authors Paul Marshall, Lela Gilbert and Nina Shea describe the kidnapping of Coptic girls for forced conversions to Islam, citing a report that documented twenty five abductions. They also point to a 2010 letter written by 18 bipartisan members of Congress to the State Department concerning allegations that Coptic girls were being subjected to “fraud, physical and sexual violence, captivity, forced marriage, and exploitation in forced domestic servitude or commercial sexual exploitation” and that financial benefits were being granted to those who forced conversion of the victims.^10^

Of course, where the law favors some, it cannot be relied on to protect anyone. The Australian Government’s 2010 Refugee Tribunal Report cited evidence that *both Coptic Christian students and Muslim Brotherhood students* were the subject of regular discrimination and mistreatment by government authorities. 11

In spite of the rise of Islamist extremism in Egypt, the majority of Muslims in Egypt were moderate compared to the extremists. They longed for political and economic reforms which would allow them more opportunity and a better life. Indeed, in the lead up to the revolution, Muslim youths, Christian youths, the educated class, women and secularists were all fed up with the lack of political freedom and the economic stagnation that resulted from the Mubarak government’s authoritarian policies. Given the wave of discontent throughout the Arab world, and given the inherent dangers to Christians in any revolutionary situation, it would have been an excellent time for the US government to take a principled stand for religious and political freedom. Instead, the administration immediately halved the money for democracy promotion in Egypt and slashed civil society funds 70%.

What of the Muslim Brotherhood, the organization which would seize the upper hand in the new Egypt, and the organization with which the Obama administration has so positively and actively engaged? An honest assessment leads to this conclusion: There was reason to engage with the Brotherhood since they were more moderate than other organized Islamist/Egyptian groups. There was also reason to fear and mistrust the Brotherhood and to put all kinds of provisos on any support we gave them. On the positive side, unlike the more radical Salafists, the Brotherhood had eschewed violence as a way of achieving its goals. Revolution against Mubarak, for the Brotherhood, was also revolution against the repressive political system which had prevented them from running for office and denied them political equality and opportunity. Marc Lynch asserts, “Brotherhood and Salafi-jihadist figures argued with each other constantly, denouncing each other over ideology and tactics. Lumping together the Brotherhood with al Qaeda would have been a major analytical error with serious policy consequences.”^12^ From a moral standpoint, too, there was reason for the United States to reach out to the Brotherhood. They had been targeted and persecuted by the Mubarak regime, and our own democratic principles meant that we should give them a chance.

But, both the long-term goals of the Brotherhood, and the ideological trajectory of the Brotherhood at the time of the Arab Spring should have given the United States major concerns about what the Brotherhood would do when and if it actually came to power. (The rising persecution and harassment of Egyptian Christians at the time should have added to these concerns.) The Brotherhood has always been clear on its goal of a global Muslim caliphate in which the Quran is the source of all law. Its slogan is “Islam is the Solution.” Former Ambassador to Egypt Daniel Kurtzer asserted, “The Muslim Brotherhood since its founding in 1928 has had one goal, and one goal only. And that is to establish an Islamic state in Egypt as a precursor to an Islamic state throughout the region. …They are very flexible on tactics. And I think we need to be careful not to mistake their tactical flexibility for their long-range strategic goals.”^13^ Although less radical than Brotherhood offshoots that sought violent means to Islamic ends, the Egyptian Brotherhood had not wavered from commitment to Sharia Law nor from its refusal to recognize Israel’s right to exist.

Moreover, although it was *less radical*, it was still ideologically extreme. It imposed restrictive dictates on women, made racist declarations about Christians and Jews, insisted that the Coptic population was much smaller than it was, and saw political institutions as vehicles for Islamization. In a 2005 interview with the newspaper *Azzman*, Mohammad Habib, key member of the Brotherhood’s highest official body, the Guidance Council, stated, “The Muslim Brotherhood rejects any constitution based on secular and civil laws, and as a consequence the Copts cannot take on the form of a political entity in this country. When the movement will come to power, it will replace the current constitution with an Islamic one, according to which a non-Muslim will not be allowed to hold a senior post, whether in the state or the army, because this right should be exclusively granted to Muslims. If the Egyptians decide to elect a Copt for the presidential post, we will issue a protest against such an action, on the basis that the choice should be ours.” 14

In addition, the Muslim Brotherhood had taken a *more* radical turn by the time of the Arab Spring, and was marginalizing and forcing out the small group of young reformers who were more open-minded and pragmatic than the majority. In 2008, hardliners were declared the winners in all five seats being contested in elections to replace empty seats on the Guidance Council. The next year, the most prominent reformist member, Abdel Monem Abou el-Fotouh, and Mohammad Habib, who had recently softened his position regarding the Copts, lost their seats, while the conservative but conciliatory Mohammed Mehdi Akef stepped down. Over the next few years, many more reformist leaders were excluded from positions of influence, or left the organization. By the time revolution broke out, the Muslim Brotherhood was dominated by ideologues who had neither desire for nor experience in forging democratic compromises.

It is inconceivable that the US government would not know that an Egypt ruled by the Muslim Brotherhood would pose a threat not only to Christians, but to any Egyptians who didn’t go along with the group’s religious-political vision. As Magdi Khalil put it after the Brotherhood won 88 seats in post-revolutionary parliamentary elections, “Actually, the Copts are not the only ones to have serious misgivings about the latest developments in Egypt’s political life: women, liberals, civil society supporters, leftists, and other advocates of democracy share the same sentiment. The champions of civil society are haunted by the nightmarish vision of religious government, and it goes without saying that the Copts, as a religious minority, are particularly concerned about the Muslim Brotherhood’s agenda with regards to the creation of an Islamic state and Islamic nationalism. … Most importantly, the Muslim Brotherhood’s history, actions, website, statements and newspaper articles confirm the intent to establish a state that has a religious nature and not a civil one.”^15^

So too, it is inconceivable that the US government was simply naïve about Mohamed Morsi’s ultimate goals when he ran for president. In his years as a parliamentarian, from 2000 to 2005, Morsi sought to make civil society, the state and the private sector more in accord with the Quran’s principles. He was elected by the Brotherhood’s Guidance Council to be the first president of the Freedom and Justice Party, which was founded in 2011. While serving in this capacity, Morsi did, in his willingness to work with other groups, reveal the “pragmatic” side that the Obama administration praised. However he also revealed his dogmatic side, as he stated that the “two-state solution is nothing but a delusion concocted by the brutal usurper of the Palestinian lands” that it was "insulting" to suggest that damage from aircraft collision brought down the World Trade Center and that no evidence had identified Al-Qaeda terrorists as the “real culprits.”^16^

A *USA Today* article entitled “Egyptian President’s Aims Unknown” published just after Morsi won the presidential election, cited a cross-section of expert opinion and was picked up by newspapers around the world. Keeping in mind that, if *USA Today* had access to this opinion, the US government certainly did as well, the article is worth excerpting:

“But his years spent studying in America have not dissuaded him from the most doctrinaire beliefs of the Muslim Brotherhood, which has called for religious law, segregation of the sexes and scorns the influence of the West and Israel, experts say. ‘If you look at his public statements over time, he tends to say provocative things about the U.S. and Israel,’ says Shadi Hamid, an expert in Islamist political parties at the Brookings Doha Center. … As a leader of the [Muslim Brotherhood] movement, Morsi is a firm believer in *sharia,* or Islamic law, as he made evident on the campaign trail. … ‘He was a loyal brother, an enforcer.’ … He is ‘an icon of the extremists in the (Muslim Brotherhood),’ says Eric Trager, an analyst with the Washington Institute for Near East Policy who is in Cairo. Trager says Morsi rose to the top of the Brotherhood's cult-like hierarchy by adherence to dogma at each level of his ascent. He is one of the main authors of the group's 2007 platform that said women and Christians should not be able to run for president, a stand that was later dropped, he says. Morsi is not likely to make serious concessions to liberals and Christians despite promises to do so and will not give them positions of real power, Trager says.”^17^

Clearly, there was no reason for the US government to be sanguine regarding Egypt’s political outcome, and many reasons for the United States to be leary. The Obama administration was privy to very troubling information about the Muslim Brotherhood and Mohamed Morsi himself. At best, its assessment of the group and of the group’s leader should have been very mixed.

Nevertheless, even before the revolution provided the Brotherhood with vastly enhanced status and unprecedented opportunities, the White House treated the Brotherhood warmly rather than cautiously, and as a mainstream political party rather than as a controversial, or at least suspect, political/religious group. In, 2009, the White House invited the Islamic Society of North America to Obama’s inauguration even though the Justice Department had blacklisted the Brotherhood as co-conspirator in the Holy Land Trial. Obama invited the Egyptian Muslim Brotherhood to his Cairo Speech as “special guests,” infuriating Mubarak. Obama was urged by Egyptian Copts to mention their plight in his Cairo Speech. But, he devoted only half a sentence to their situation, while making a strong and repeated case for tolerance of Islam. He appointed a Brotherhood-tied Islamist, Rashad Hussain, as US envoy to the Organization of the Islamic Conference and, in 2010, sent Hussain to Egypt to meet with the Brotherhood’s Grand Mufti.

It is significant that secularists and Christians in Egypt received no comparably supportive signals from the administration.

## Revolution

Protestors in Tahrir Square and other sites across Egypt demanded political reform, an end to the hated emergency law and free and fair elections. Different groups and individuals, including the Muslim Brotherhood, moderate Muslims, secularists, many women and many Christian Copts came together in support of this cause. There were even signs of unity and support between Brotherhood protestors and Coptic protestors. As Vivian Ibrahim puts it, “As images of Tahrir Square were beamed via satellite and the world watched a ‘televised revolution,’ the iconic images of national unity were brought to the fore. Copts stood guard over Muslims as they performed Friday prayers in the square, while Muslims in return stood guard over Coptic Sunday mass.”^18^

The April 6 Movement, which initiated the revolt in Cairo, made explicit demands regarding democracy and human rights. Although some are already re-writing history by defining the uprisings that began in Tunisia in terms of Islamic-extremist goals, this is a flawed depiction. Historian James Gelvin delineates distinctions between the goals of the Arab Spring and the goals of organizations like al Qaeda:

“Although protesters in various countries found inspiration and learned from protests elsewhere in the Arab world, each uprising was a national uprising, targeting a specific government against which protestors held specific grievances. Al-Qaedists believe that the Crusader-Zionist conspiracy against Islam obligates every Muslim to engage in ‘defensive jihad,’ which, for them, means armed struggle. Yet from Tunisia to Egypt to Bahrain, protesters embraced the tactic of nonviolent resistance. Finally, al-Qaedists believe that Muslims should obey the rule of God, not the rule of man, and that true freedom lies in obedience to Islamic law and freedom from the materialism and oppression of the West. Yet the central demands of the protesters include democratic governance–rule by the majority, not by the word of God–and respect for internationally accepted norms of human rights. These are certainly not al-Qaeda’s ideals.” 19

Daniel Byman notes that the Arab uprisings actually removed one of Al Qaeda’s reasons for being: “When dictators reigned supreme in Arab lands, al Qaeda could score points by denouncing despotism–Zawahiri even wrote a book condemning the crimes of Mubarak. When dictators such as Mubarak fall to pressure from pro-democracy protestors, however, al Qaeda loses one of its best recruiting pitches: the repression Arab governments inflict on their civilians.” 20

Still, there was reason to fear that the Egyptian revolution, like so many before it, would take a radical turn. There are two things about the Egyptian revolution that those inside the US government would have immediately seen that the rest of us did not. First, the Egyptian military had a vested interest in the revolution because the military was vehemently opposed to succession of power to Mubarak’s son Gamal, who was promising to free the economy from military control. The military influenced all aspects of Egyptian society, including the economy, and its oligarchical power was threatened by the prospect of reform. Second, although the revolution included various parts of Egyptian society, including youths eager for western-style freedom, the best organized among the parts was the Muslim Brotherhood. Evidence indicates that most Brotherhood demonstrators were genuine in their desire for free elections. On the other hand, their philosophy and history were cause for concern over how committed to reform they would be once they achieved a higher place in Egyptian politics and society.

After the Revolution, the Egyptian military and the Muslim Brotherhood were, in the words of *Stratfor Intelligence*, “savvy powerbrokers” in that they were careful not to pose too great a challenge to each other, since they both had an interest in marginalizing other groups.^21^ The military utilized recurring street violence to “divide and conquer” and to keep the army’s position strong, while the Brotherhood kept its own people off the streets in those cases where a military crackdown on protests worked to its advantage, in that it intimidated those looking for *a different kind of change* in Egyptian society. The military, in turn, permitted elections in which the Muslim Brotherhood was fully accepted as candidates. No wonder Iran, in spite of the divide between Shiite (Iranian) and Sunni (Egyptian) Muslims, immediately, chillingly, praised the Revolution, predicting that it heralded an Islamic Middle East.

Given all of this–given the power vacuum created by the revolution, given Iran’s overt eagerness regarding the outcome, given that Egypt was the United States’ best Middle Eastern ally apart from Israel, and that that alliance was essential to Israel’s existential concerns, given the perilous position of Christian Copts, women and secularists in any Brotherhood-dominated government, it behooves us to ask: Why didn’t the Obama administration put more effort into encouraging democratic reform and moderate political forces before the revolution occurred? Why did President Obama, after days of indecision over whether to support “stability” via the maintenance of Mubarak or the “stable transition” to a post-Mubarak government, then decide to insist that Mubarak step down immediately? Why did Mubarak have to “go now” if the much worse Ahmadinejad, and the much worse Bashar Assad, in the face of a much more widespread collapse of support, did not?

Why didn’t the administration use our substantial leverage to influence the rebellion, once it ousted Mubarak, toward continued friendly relations with the United States, *genuine* democratic reforms and continued support of the peace treaty with Israel? After all, the United States routinely gives Egypt two billion dollars a year most of which goes to military and technical aid, and the rest of which goes to humanitarian assistance.

Instead, as the revolution heated up, and *before* Mubarak’s exit, the administration volunteered that it “accepted” and “welcomed” the Muslim Brotherhood’s participation in Egypt’s “political dialogue.” The White House also declared that *all opposition groups* (including the Muslim Brotherhood) should be represented in the post-Mubarak government. In an *NPR* interview in January, Secretary Clinton said it was in the “best interest” of the United States to have “more democracy, more openness, more participation.” She also said, “Today, we learned that the Muslim Brotherhood has decided to participate, which suggests that they are now involved in the dialogue that we have encouraged.^22^ This positive stance toward the Brotherhood was new for the United States and was big news. Headlines across the world read, “Clinton Welcomes Muslim Brotherhood Participation.” It was one thing to welcome Brotherhood participation after the fact; it was another to actively encourage it beforehand.

In an article that is no longer available online, the NY Times reported on January 4th that the administration was seeking “close ties” with the Muslim Brotherhood, correctly adding: “The administration’s overtures—including high-level meetings in recent weeks—constitute a historic shift in a foreign policy held by successive American administrations that steadfastly supported the autocratic government of President Hosni Mubarak in part out of concern for the Brotherhood’s Islamist ideology and historic ties to militants.”

Brotherhood participation in presidential elections was so controversial in Egypt itself that, even after Mubarak’s ouster, Brotherhood leaders *themselves* promised not to run for election. *Rather than attempting to publicly hold them to this pledge, or at least staying neutral on the subject, Obama and Clinton again publicly offered support for Muslim Brotherhood participation in Egyptian elections.* “It’s in our interests to engage with all of the parties that are competing for parliament or the presidency” a senior U.S. official told *Reuters*, on condition of anonymity.^23^ Emboldened, the Brotherhood formed the Freedom and Justice Party for upcoming elections with Dr. Saad Ketatni as its leader. The new party’s spokesperson noted that “when we talk about the slogans of the revolution–freedom, social justice, equality–all of these are in the Sharia.” (Islamic Law) Even after all of this, however, Egyptian Brotherhood leaders reiterated that they would not run a candidate for President and would not compete for more than a third of the seats in a new parliament.

Adding to the supportive signals the Brotherhood received from the US administration, the day before Mubarak resigned, the White House sent Intelligence Czar James Clapper to Congress to testify that the Brotherhood is a “moderate” and “largely secular organization” that has “eschewed violence” and has “no overarching goal, at least internationally.”^24^ As if to publicly dispute Clapper’s claims, the Brotherhood’s *spiritual* leader Sheikh Yusuf Qaradawi was given a hero’s welcome in Tahrir Square. Further, the Brotherhood vowed to tear up Egypt’s thirty year peace treaty with Israel and quickly worked to formally reestablish Egyptian ties with Hamas. The timing was strange, then, when President Obama publicly demanded that Israel relinquish land to the Palestinians, and the Justice Department announced that there would be no further prosecution of Muslim Brotherhood front groups for funneling millions to Hamas. In a break from U.S. tradition, the administration announced in June that it would formally recognize and resume diplomatic contact with the Muslim Brotherhood.

Is it mere coincidence that Brotherhood activists were emboldened? (History reveals again and again that aspiring extremist leaders opportunistically exploit the friendly gestures and conciliatory signals of democratic powers.) Also in June: Nina Shea reported in the *New Republic* on “a reinvigorated effort by some of the country’s more radical Islamists to establish Egypt’s identity as a thoroughly Islamicized and Arabicized state” and noted “a heightened campaign of violence” against the Copts. Shea feared this would led to “a mass exodus from the country-an event which, if it transpires, will have devastating effects on the multicultural makeup of the entire Middle East.”^25^ In reference to the Coptic Bishop’s public attention to the problem, Shea urged, “We should heed the Bishop’s cry for help before it’s too late.” Shea was right. Shortly after the Revolution, incidences of violence toward and bullying of Christians increased dramatically.

Adding further to the supportive, emboldening messages the Brotherhood received from the US, a number of meetings between US Ambassador Anne Patterson and Brotherhood members ensued shortly after the Revolution. The ambassador seemed to favor the Brotherhood and hardline Salaafists over the secularists and Christians. In fact, she is said to have turned down requests from heads of political parties and other secularist politicians. Deputy Secretary of State William Burns and Senator John Kerry sent similarly supportive messages by visiting Muslim Brotherhood headquarters and talking with one of their revolutionary leaders, Khairat El Shatar, whom Kerry went so far as to publicly praise. Note that these meetings occurred *before* the Muslim Brotherhood had emerged as the leader in post-revolutionary Egypt.

Frida Ghitis reported upon a turning point in August of 2011. Tahrir Square “became the scene of a stunning change at the vanguard of the revolution, when Egypt’s Islamists shed their cloak of unity with secular liberals and declared their intention to pursue their own agenda:”

Leaders of the April 6th movement, religious parties, and leftist groups had agreed to a demonstration of solidarity without religious banners or slogans that might divide them. But liberal groups were dumbfounded when the Muslim Brotherhood and Salafist groups started unfurling banners denouncing secularism and calling for religious law. … Days later, in actions one liberal blogger called ‘our Kristallnacht,’ the young idealists suffered another painful blow. When they least expected it, the army went after their last remaining sign of strength, a tent city at Tahrir. … When hundreds of soldiers started beating the protestors and shooting in the air, large crowds of Egyptians joined them, chanting, ‘Allahu Akbar.’^26^

The Supreme Council of the Armed Forces, at first praised for relative restraint as it assumed control of the post-Mubarak transition government, after the crackdown in April, continued to brutally attack demonstrators. In addition, it prosecuted regime critics in military tribunals, sexually assaulted female protestors and maintained the hated emergency law. In October, 2011, state action took an explicitly anti-Christian turn. When Copts staged a peaceful protest in reaction to the torching of a church and other shows of intimidation, the military response was cruel and brutal. According to Copts themselves, who produced photographs to prove it, a massacre ensued. Muslims who joined the demonstration “seemed to split between those who sided with the military and those who tried to shield the Christians.”^27^

As the military used force to intimidate civilians and Islamists demonstrated their release from Mubarak-era restraints, the position of Christians deteriorated rapidly. But the White House continued to describe violence against Christians as “sectarian violence.” At no time did President Obama, Secretary Clinton, Secretary Panetta or other members of the Obama administration take a forthright, unambiguous stand for a truly free and democratic transition in Egypt that included human rights, religious liberty, civil society and the impartial rule of law.

On Copticliterature.wordress.com, we find an outcry not only against the brutality of the new Egyptian regime, but also against the small-hearted response by the US and British governments, particularly in regard to the Maspero Massacre of October, 2011: “But while many in the West were deeply troubled and wanted their governments to act by protecting the Copts of Egypt, some of the responses that we saw coming from the West’s center of power failed to respond to the people’s concerns. The responses of Britain and the United States of America were particularly troubling. Their politicians, sadly, did not match their nations in their outrage as newswires cabled and reported the bloody Sunday in Cairo. What did President of the United States of America–that great country which the Copts love and look for as leader of the free world–had [have] to say about the massacre of the peaceful and unarmed Copts by Egypt’s army? I will simply copy the White House statement on the event (it calls it ‘violence’ and does not mention the word massacre.)”^28^

In a distressing addition to multiple instances in which President Obama has described the slaughter of innocents as “violence” between two flawed sides, the White House stated in a memorandum following the massacre: “The President is deeply concerned about the violence in Egypt that has led to a tragic loss of life among demonstrators and security forces. …Now is a time for restraint on all sides so that Egyptians can move forward together to forge a strong and united Egypt.”^29^ The memorandum went on to state the “belief” that the rights of minorities “including Copts” should be respected, but ended by giving Prime Minister Sharaf credit for calling for an investigation and appealing to “all parties to refrain from violence.”

This moral equivalence and indifference to human suffering–this verbal gift to tyrants by way of calling murderous crackdowns on peaceful protestors “violence”-is an affront to the principles that the United States, at its best, stands for. Where was the outcry from American citizens in response to this degradation of the American tradition? Yes, there was some passion in response to the harassment and detention by the Egyptian government of our own citizens working for NGOs. But, where was the passion for our fellow human beings? It would be nice if our leaders encouraged, rather than discouraged, the oft-observed American concern for the world’s oppressed.

As more violence against Christians occurred-including the burning of churches, sexual harassment of women and girls, bullying on the streets and forced confessions for crimes not committed-our President and Secretary of State stayed mostly silent.

## The Muslim Brotherhood Reveals its True Aims

In January 2012, Islamists (the Muslim Brotherhood and the more extreme Salafists) won 72% of the seats in the lower house of Egypt’s Parliament. The Brotherhood won 235 out of 498 parliamentary seats. Also in January, the Brotherhood’s deputy leader Dr. Rashad Bayoumi said the Brotherhood would not recognize Israel “under any circumstance,” would “never” negotiate with Israelis, and would take legal procedures towards canceling the peace treaty.^30^ In spite of this, White House press secretary Jay Carney urged listeners not to “judge the disposition” of a government and parliament that was only “just beginning to take shape,” while Hillary Clinton emphasized that “what parties call themselves is less important than what they do.” What was important, she said, was the Brotherhood’s adherence to democratic procedures. State Department spokesman Mark Toner stated, “We’ve been very clear about how we view the Muslim Brotherhood, which is that if they’re committed to the democratic process, we welcome them as part of the political process.”^31^

Coptic Christians and Egyptian secularists were neither as hopeful, nor as welcoming. Avi Asher-Schapior reported, “Although Egypt’s nascent democratic institutions inspire little confidence …Coptic groups have directly petitioned Egypt’s parliament, completely bypassing the clerical hierarchy. In a caustic letter addressed to the Muslim Brotherhood’s parliamentary majority last January, the Union of Maspero Youth questioned the ruling Islamists’ commitment to equality: ‘Will you rule us according to law or will you deal with us as a minority and Christians and how will you deal with our women and how will you see their faces and their uncovered hair?’ the letter read. As the confrontational tone of the letter suggests, far from feeling represented by Egypt’s first democratic parliament, many Copts view the institution with open hostility.”^32^ But, as Shadi Hamid has shown, “Egypt’s fractious opposition spent little time thinking seriously about what it would do in power” thereby allowing the more organized and prepared Muslim Brotherhood to mobilize.

Behind the scenes, American policies went even further than public statements in legitimizing the Muslim Brotherhood and boosting their status. *Front Page Magazine* broke the story that the State Department’s Special Coordinator for Middle East Transitions William Taylor–and his office–had been giving Egyptian Islamists training to prepare for the election contests that began on November 28th. Taylor justified this by saying assistance was available to all parties and that “sometimes Islamist parties show up, sometimes they don’t.”^33^ In addition, the Department of Homeland Security issued new guidelines that empowered Brotherhood members as interlocutors with Muslims in the United States and required FBI training materials to be approved by Muslim “community leaders” and “interfaith groups.” The White House issued a “National Strategy for Empowering Local Partners to Prevent Violent Extremism” that effectively guaranteed the Muslim Brotherhood a say in its policies. Deputy Secretary of State Bill Burns met with Mohamed Morsi in Washington, indicating high-level outreach to Islamist leaders. Morsi welcomed the meeting and called on Washington to adopt a “positive position concerning Arab and Muslim causes.”^34^

Hillary Clinton described all of this as “re-engagement in light of Egyptian developments.” According to the *New York Times*, “Mr. Obama told aids he was impressed with the Egyptian leader’s [Morsi’s] pragmatic confidence. He sensed an engineer’s precision with surprisingly little ideology.”^35^ President Obama and Secretary Clinton took this stance even though the marriage of convenience between the Egyptian military and the Muslim Brotherhood was already in evidence, as was the Brotherhood’s Islamist, anti-Christian agenda. By January, 2012, the military junta and Muslim Brotherhood had formed a power-sharing agreement. Amnesty International’s Middle Eastern director Hassiba Hadj Sahraoui intoned, “That the Minister of Justice could now give an army responsible for killing, torture, and thousands of arbitrary arrests and unfair trials the power to arrest and detain civilians beggars belief. It is nothing less than legally sanctioning abuse.”^36^

Carolyn Glick’s report on the implications of the agreement, and on the repression of Christians and women in the new Egypt, is enlightening:

This is bad news for women and non-Muslims. Egypt’s Coptic Christians have been under continuous attack by Muslim Brotherhood and Salafist supporters since Mubarak was deposed. Their churches, homes and businesses have been burned, looted and destroyed. Their wives and daughters have been raped. The military massacred them when they dared to protest their persecution. As for women, their main claim to fame since Mubarak’s overthrow has been their sexual victimization at the hands of soldiers who stripped female protesters and performed ‘virginity tests’ on them. Out of nearly five hundred seats in parliament, only 10 will be filled by women. The Western media are centering their attention on what the next Egyptian constitution will look like and whether it will guarantee rights for women and minorities. What they fail to recognize is that the Islamic fundamentalists now in charge of Egypt don’t need a constitution to implement their tyranny. All they require is what they already have–a public awareness of their political power and their partnership with the military.^37^

On April 14, 2012, Egypt's high election commission disqualified 10 of the 23 presidential candidates on various grounds, leaving Egyptians with thirteen presidential candidates. Unfortunately, many moderates and Christians vetoed the elections in protest of a post-Mubarak system they believed had already gone astray. The two highest vote receivers, who would then face each other in elections, were a member of the Muslim Brotherhood (Mohamed Morsi), and Hosni Mubarak’s last prime minister, who was part of the military establishment.

Muslim Brotherhood leaders had themselves offered and pledged not to run a candidate for president. But that was according to the old rules–under which a party that described the destruction of Israel and the institution of Sharia Law as goals was viewed with alarm. *Obama and Clinton themselves threw out the old rules.* No wonder the Brotherhood ignored its pledge and ran a candidate for the presidency. Who ran and won? Mohamed Morsi, who had been so generously greeted in Washington, and whose “pragmatism” had been praised by the president himself.

While some groups boycotted the presidential election due to the collaboration between the hated military and the Brotherhood in the run-up to the election, due to revelations about corruption in the parliamentary elections, and due to discontent with the choices available, Brotherhood activists refused to boycott the election. Reported Richard Spencer from Cairo, “The group, which won the parliamentary elections that have now been ruled invalid, issued a statement saying Egypt was facing a ‘counter-revolution plainly witnessed by all.’ … Many revolutionary activists are calling on voters to spoil their ballots as a protest against what they see as manipulation of the election.”^38^ Reported Sarah El Deeb of the *Associated Press*, “Whoever wins after two days of voting, Egypt’s military rulers will remain ultimately at the helm, a sign of how Egypt’s revolution has gone astray 16 months after millions forced Mubarak to step down in the name of freedom …we are forced to make this choice. ‘We hate them both’ said Sayed Zeinhom at Cairo’s Boulak el-Dakrour … .”^39^

In his first public speech as President, on June 24, 2012, Morsi provided reason for both hope and fear. He said, "We came to the world with a message of peace. We will maintain international charters and conventions and the commitments and agreements Egypt has signed with the world. … We will also work to make the Egyptian system of ethics… in addition to human values particularly in freedoms, respect for human rights, maintaining rights of women and children." He praised “God's help and these sacrifices, the precious blood of our honourable martyrs and our great injured men." He also praised the military: "I love them [soldiers] and appreciate their role and show keenness to strengthen them."^40^ Like most would-be dictators, he claimed he stood for *unity* and urged his countrymen to put aside their differences. “"I invite you, the great Egyptian people… to cement bonds amongst us, to strengthen our comprehensive national unity. …This national unity is the only way to get Egypt out of this difficult crisis.”

As for the marriage of convenience between the Muslim Brotherhood and the military, it was less necessary for both groups once they had succeeded in marginalizing moderate reformers. After the presidential election, the Brotherhood and the military at times collaborated, and at times struggled against each other for power. Initially, the reformist groups that the revolution had left behind wanted nothing to do with either. As Shadi Hamid put it, “In the new Egypt, the military and the revolutionaries quickly found they wanted different things. The former wished to preserve stability at all costs, while the latter wished to push forward aggressively with democratization.”^41^

As presidential voting came to an end, the Supreme Council of the Armed Forces (SCAF) issued a constitutional declaration, in which it granted itself extended powers, including legislative, constitution drafting and other supervisory activities. It announced a parliamentary election to take place one month after approval of the constitution, thus attempting to unilaterally determine the issue of whether the current parliament had been legitimately elected. The SCAF had previously promised a full transfer to civilian rule after the election. Egyptians were thus faced with a new “democratic” government that was dominated by two groups (the Brotherhood and the Army) that had *already* broken major promises.

President Morsi, too, moved quickly to use his victory to maximize Muslim Brotherhood control of Egyptian government and Egyptian society. In July, he chose an unknown former water minister sympathetic to Brotherhood ideas, Hesham Qandil, as his prime minister. The Prime Minister, in turn, appointed only one Copt to his cabinet, despite the president’s previously announced commitment to include greater numbers of Copts in his government. An alliance of pro-democracy advocates called The National Front Alliance that included secularists and moderate Muslims publicly criticized Morsi’s reneging on his promise to form a “unity government.”^42^ The lack of a new constitution, and the intentional exclusion of pro-democracy groups from key leadership positions further damaged the legitimacy of Morsi’s government.

Of course, the unity Morsi praised was a pipedream, as each faction-the military, the elected Brotherhood, the Judiciary-fought for dominance. Unity would soon mean oppression of those who didn’t agree with Brotherhood-Islamist goals, and the real goals of the Brotherhood would soon become apparent. Morsi would tacitly accept Brotherhood and Salafist violence against and intimidation of Christian Copts. He would push a Constitution that declared Sharia law “the main source of legislation” and would move to undo the independence of the judiciary, which was packed with judges appointed by former President Mubarak. He would state that the peace treaty with Israel would eventually have to be “revised” while making positive gestures toward Hamas. He would issue edicts that granted him immunity from appeals in courts for any decisions or laws he declared until a new constitution and parliament were in place. Significantly, his most dramatic moves toward near-absolute power would come in November, 2012, at a time when he was enjoying abundant, unsparing praise from President Obama and Secretary Clinton for brokering a cease-fire between Israel and Gaza’s Hamas. This shows clearly that our “partnership” with the new Egypt should have been tempered with our use of strategic leverage and our advocacy of democratic principles.

Engagement with Brotherhood leaders in the spirit of respecting the wishes of the Egyptian people, in the attempt to encourage continued close relations between the United States and Egypt-or in the attempt to encourage moderation on the part of the Brotherhood-are one thing. But, the lack of demonstrated U.S. enthusiasm for human rights and individual rights, and the lack of demonstrated U.S. concern for Israel’s rapidly deteriorating position as it faced the likelihood of an Islamist Egypt should give us pause. Stated *Human Rights First* on the one year anniversary of the Egyptian uprising, “The U.S. government should now focus on delivering a sustained clear message about its policies and goals in Egypt, one that emphasizes U.S. support for civilian democratic rule. … The only way to advance democracy is by implementing the democratic process and building safeguards for democratic rights and freedoms as the process moves forward.”^43^ But, Obama and Clinton spoke up for democracy rarely, and when they did, their speech lacked direction and conviction. They did not use U.S. influence to “safeguard” democracy. Moreover, their foreign policy team focused far too little on the strategic consequences of the Muslim Brotherhood’s ascendancy.

This is not to say that the administration was wrong in attempting to forge positive relations; American policy has often included trying to nudge activist groups toward more moderate means and ends by forging ties with them. Moreover, we cannot support democracy in the Middle East without accepting that voters might very well vote Islamists into power. I was not in favor of an intractable stand regarding the Muslim Brotherhood due to concern for participatory government and due to sympathy with Brotherhood grievances under Mubarak. But, the Obama administration’s reaction to events in Egypt lacked strategic or pro-democracy direction. In the words of one conservative columnist, the approach was “apathetic” and “dilatory.” In the words of one liberal columnist, it was “uncertain” and “weak.” Especially disturbing, *the only consistent policy, before, during and after the revolution, was reaching out to the Muslim Brotherhood.*

We should not have taken our support of the Brotherhood so far, and we should have done more to support other freedom-seeking Egyptians: those who relied on the fact that power was about to devolve away from Mubarak and had placed hope in reform; those who had marched in rebellion against the regime in hopes of creating liberal democracy; those who boycotted elections and objected to the new government because they saw the entire system as rigged. As Neil Ferguson put it, “If we want to see secular-democratic forces prevail in a country like Egypt, which is overwhelmingly a Muslim country, which has a tradition of Islamic radicalism in the form of the Muslim brotherhood, it is not going to happen by itself. The lesson from Eastern Europe, going right back to the Cold War, is that the United States had to very actively support democratic forces until finally the moment came in 1989 when they could step forward into the limelight and they were ready.” Ferguson added, “We haven’t got a plan here, and if we don’t have a plan to build a secular democracy in Egypt, it’s not going to happen.”^44^

Hillary Clinton insisted that it is not “who” is in power in Egypt that matters, but “what” they choose to do that matters. By her own logic, then, why wouldn’t we have made the continuation of billions of dollars in annual aid to Egypt contingent upon the Brotherhood’s recognition of the state of Israel, the group’s discontinuation of support for Hamas, and improvement in human rights and civil liberties? Instead, in March of 2012, the White House indicated that Secretary Clinton would use her “waiver authority” to release part of the 1.5 billion in aid to Egypt, thereby bypassing a law requiring that more aid to Egypt be contingent upon Egypt’s improvement in human rights and transition to civilian government.

A statement issued by the State Department defending the move is telling: “As the Secretary’s statement makes clear, as the statement we released with regard to her decision makes clear, we have a huge number of interests and equities at stake in relationship with Egypt. This is a strategic partnership; so rather than talking about leverage, we’re talking about partnership, as we have for all these years. … We have a new Egypt emerging. So U.S. support in all of its forms–FMF, ESF, in countries around the world–is designed to allow us to support the partnership that we have with countries and the developments that we want to see in countries in a more democratic, prosperous, stable, secure direction.”

“Rather than talking about leverage, we’re talking about partnership.” That statement encapsulates the Obama administration’s approach to the Muslim Brotherhood. Before the Brotherhood won seats in parliamentary elections; before the Brotherhood broke promises that it would not run in presidential elections; before the Brotherhood consolidated power and made deals with the Egyptian military and security forces; before discrimination against the Christian Copts devolved into persecution and violence; before the emergence of a constitution that enshrined Sharia Law and mostly excluded the wishes of moderate Muslims, Christians and secularists ----- *before all of this and after all of this*, the Obama administration treated the Muslim Brotherhood as a partner.

Many in Congress expressed disappointment in the decision to unconditionally release the aid, including Democratic Senator Patrick Leahy, who stated, “I know Secretary Clinton wants the democratic transition in Egypt to succeed, but by waiving the conditions we send a contradictory message. The Egyptian military should be defending fundamental freedoms and the rule of law, not harassing and arresting those who are working for democracy. They should end trials of civilians in military courts and fully repeal the Emergency Law, and our policy should not equivocate on these key reforms.”^45^ President of *Freedom House* David Kramer chimed in, “The decision to waive the conditions, partially or in full, on military aid sends the wrong message to the Egyptian government–that U.S. taxpayers will subsidize the Egyptian military while it continues to oversee the crackdown on civil society and to commit human rights abuses. … A resumption of military aid also sends the wrong message to the Egyptian people–that we care only about American NGO workers, not about the aspirations of the Egyptian people to build democracy.”^46^ Stephen McInerney, executive director of the Project on Middle East Democracy, also criticized the decision, “particularly as Egyptian and American organizations working to support Egypt’s transition to democracy remain very much under threat.”^47^

The Obama administration’s unconditional support of the Muslim Brotherhood helped legitimize the group as the rightful representative of Egyptian people, when, actually, its popularity and place in Egyptian society was tenuous. Michael Wahid Hanna observed that the administration had given “outsized attention on the cultivation of ties with the now ascendant Muslim Brotherhood, often heedless of broader Egyptian political dynamics.” He went on, “The United States cannot micromanage Egyptian politics, but it retains real influence and it can, at the very least, attempt to staunch negative trends as opposed to reinforcing moral hazards. The current Egyptian government now believes in its own centrality and strategic significance, and it further believes that it has the uncritical support of the United States and the international community.”^48^

Why would President Obama and Secretary Clinton *not* take a clear stand for human rights and the rule of law in Egypt, and why would they *not* use financial leverage to press for improvement in these matters? I use the word *clear* because Clinton did *on occasion* defend rights in Egypt and elsewhere. Such was the case in her statement upon the release of the State Department’s Report on International Religious Freedom in September, 2011. She said: “Hatred and intolerance are destabilizing. When governments crack down on religious expression, when politicians or public figures try to use religion as a wedge issue, or when societies fail to take steps to denounce religious bigotry and curb discrimination based on religious identity, they embolden extremists and fuel sectarian strife. And the reverse is also true: When governments respect religious freedom, when they work with civil society to promote mutual respect, or when they prosecute acts of violence against members of religious minorities, they can help turn down the temperature. They can foster a public aversion to hateful speech without compromising the right to free expression. And in doing so, they create a climate of tolerance that helps make a country more stable, more secure, and more prosperous.”^49^ Clinton was careful to couch her plug for religious freedom in terms of its practical benefits (stability, security, prosperity); as usual, she was careful not to take an overt moral position.

Such statements were welcome exceptions to the administration’s general stance of impassivity, but troubling questions remained. Here’s a particularly troubling question: Why has the administration been mostly silent and indifferent about the persecution of Christian Copts, secular-reformist groups and women in the new Egypt? By April, 2012, Egypt’s Coptic Orthodox Church was announcing its withdrawal from the constitutional assembly’s drafting of a new constitution, saying its domination by Islamists made Coptic participation “pointless.” Other liberal parties joined in announcing their withdrawal from the assembly, seeing their hopes for representative government as already lost. Given the administration’s early support for Brotherhood “participation” in Egypt’s government, this would have been a good time to pressure the Brotherhood to make *participation* palatable to Christians and others who had been excluded and/or were threatened by the new power structure. Again, by the Clinton’s own logic-if it is not who rules but what they do that matters-why the silence in response to what Morsi and the military have “done?” Why the non-response to the cries of the oppressed for US support?

Not surprisingly, when Secretary Clinton visited Egypt in July 2012, she was met with widespread protest from Christian Copts and secular activists, who objected to what they believed was the administration’s role in helping the Muslim Brotherhood consolidate power. Citing a stream of meetings between high-level administration officials and Muslim Brotherhood leaders, Egyptian-American human rights activist Michael Meunier explained frustration over US policy: “The MB used these high-level meetings to tell the Egyptian people that the U.S. is supporting them and does not object to their rule. Many of us reached out to US officials at the State Department and complained that the US policy regarding the MB was putting the secular forces in Egypt at a disadvantage because it seemed to be propping the MB, but our concerns were dismissed. We warned of the MB’s desire to impose Sharia law once in power and the grim effect it would have on the rights of the millions of Christians and moderate Muslims, and on women and children, yet all our warnings were dismissed.”^50^

Human rights groups estimate that more than 100,000 Copts have left Egypt since the revolution.^51^ Many Copts have been denied property rights, and protection of the law. Coptic-owned businesses have been harassed, while Christians report that they have been taunted and maligned, and sexually targeted. There has been a dramatic upsurge in assaults on Copts and attacks on Coptic churches, and the government and military chose to look the other way. No wonder the US Commission on International Religious Freedom asked the State Department to place Egypt on its list of “countries of particular concern” regarding the egregious violation of religious freedom. The State Department declined, saying it prefers to work with the Egyptian government to improve conditions for Christians.

Speaking at the Carnegie Endowment for International Peace on July 30, 2012, Clinton did indicate, however, that she was aware of the Egyptian Christian’s plight: “I heard from Christians who want to know that they will be accorded the same rights and respect as all Egyptians in a new government led by the Islamist party. … They wonder, will a government looking explicitly to greater reliance on Islamic principles stand up for non-Muslims and Muslims equally. Since this is the first time Egypt has been in this situation, it’s a fair question.”^52^

Indeed, it was a fair question, which should have led to another: What could the United States do to encourage a more positive outcome?

## Egypt Steps Up Repression

Those Egyptians who had hoped the new Constitution would secure more freedoms and lead to civil society were to be disappointed. The problem was not just that the Constitution itself did not adequately protect rights of minority groups and individuals. The problem was that, in the spirit of all would-be dictators, President Morsi issued extra-constitutional decrees, placed restrictions on the press, clamped down on the opposition, and discriminated against and marginalized certain groups. Pressure upon Egyptian journalists, Christians, secularists and human rights groups, combined with pressure upon NGOS working within Egypt for democratic reform quickly signaled that America’s “outreach” to Morsi did not translate into improvements in the lives of most Egyptians, nor into the strengthening of US/Egyptian strategic ties. When threats to our NGO workers were ultimately resolved with their release, the administration took no stand for the Egyptians themselves who were still facing criminal charges for their association with the NGOs.

In what appeared to be a positive breakthrough on the geopolitical front, in November, 2012, Morsi helped broker a ceasefire between Israel and Hamas. He received lavish praise from President Obama and Secretary Clinton for doing so, with Clinton lending the occasion visibility with a brief stop in Egypt for personal talks with the Egyptian president. However, as Dennis B. Ross and James F. Jeffrey of the Washington Institute observed in their Strategic Report, “[But] Morsi’s behavior domestically the day after the ceasefire should again remind us of his basic purpose and orientation: he immediately sought to parlay his role in the ceasefire and the international plaudits he won for it by removing all judicial oversight on his exercise of power.”^53^ The fact that Morsi made this power grab just after personal conversations with Hillary Clinton, and that the administration did not criticize him for it, were also painful reminders that the administration’s policy favored “regional stability” over human rights, and did not, in the spirit of our best foreign policy traditions, work for both.

In spite of the huge risks incurred by expressing opposition to the increasingly repressive government, Egyptians took to the streets en masse in November, 2012, to protest rigged elections, the opaque process of writing the Constitution, the assault on the judiciary and its rulings and the government’s failure to institute genuine democratic reforms. After a night of street fighting between Morsi supporters and secular opponents left at least 6 dead and 450 wounded, nine Morsi administration officials quit in protest. One of those who quit was the new general secretary of the commission overseeing the planned referendum. “I will not participate in a referendum that spilled Egyptian blood,” he said.^54^

In December, Coptic Nationalists announced that they would boycott the referendum on the constitution, calling it “an Islamist constitution and not an Egyptian constitution” and saying there were no assurances of “fairness and transparency” in the referendum. Noting that the Brotherhood’s and Salafists votes in previous elections were “magnified by those non-Islamist Egyptians who saw at the time that Morsi was the lesser evil compared to Shafi, who was seen as part of Mubarak’s regime,” they declared: “The situation has now changed: the dictatorial nature of the Islamists has been revealed to millions of Egyptians; their incompetence in managing the affairs of the country has been remarkable; and the economic situation is deteriorating while the country is approaching complete collapse.”^55^ It is telling that while President Morsi would concede *part* of the decree that removed judicial restraints on his power, he refused to delay the referendum on the constitution for the sake of promoting the “unity” he supposedly wanted.

Strikingly, the United States agreed to send F-16 s to the Egyptian government and to give Egypt additional aid at precisely this time of growing fallout-when Egyptians were taking to the streets en masse in protest. The message was clear: US support of the Morsi government remained unconditional. Egyptians were thus indirectly pressure by the United States itself to get behind the government and to tone down their expectations. Discussing the US decision to deliver the advanced aircraft to the Egyptian military, retired Brigadier General Safwat Al-Zayat told *Al-Ahram Weekly*, “It is obvious that the finalization of the deal on 11 December, which happened to be at the height of the mass demonstrations in Tahrir Square against Morsi, conveyed a political message. Between the lines, Washington was sending a message to three parties. The first was to Morsi and it states, ‘We support you. Move ahead.’ The second was to the army and it said, ‘We are encouraging this man,’ meaning Morsi. The third was to the opposition and is said the same thing.”^56^

With most Christians and secularists having resigned in protest, the constitution passed with 63% of the vote after its second referendum on December 22. Protests erupted again, this time against the rushed and distorted process which had enabled an Islamist-oriented constitution. *Youtube* videos captured gangs of men loyal to the Muslim Brotherhood beating and assaulting protestors. Around the country, Coptic Christians were being bullied, intimidated and even raped, with immunity. Sharia Law was being instituted, step by step.

Significantly, however, the US administration linked its support for a massive new infusion of financial aid to cooperation with the International Monetary Fund *instead of* with progress on religious freedom and human rights. Not only that, the new loan was supported *in spite of* Morsi’s Islamization of Egyptian government and society. The *Washington Post* reported on January 6, 2013, “Egyptian President Mohamed Morsi rebuilt his cabinet Sunday, replacing 10 ministers and amplifying the Islamist presence in the government. The move, in which at least three Islamists were appointed to major economic ministries, comes a day ahead of a planned visit by a top International Monetary Fund official to discuss an impending 4.8 billion loan. …. Islamist political parties gave their support to the latest move, but some opposition members criticized it, saying it served only to further consolidate Islamite control of top government positions weeks after a conflict over the religious character of Egypt’s new constitution left the country bitterly divided.”^57^

When, shortly thereafter, a video was released showing Morsi spewing anti-Semitic rhetoric, Shoshan Bryen of the Gatestone Institute had this to say: “The Obama administration placed a very heavy bet on its ability to manage relations with Morsi, and the world’s discovery of his virulent anti-Semitism will not change it. Key to ‘managing relations’ with Morsi is ignoring almost everything related to the Muslim Brotherhood and everything Morsi does that defies democratic norms. This includes ignoring the Brotherhood’s lie that it would not run candidates for all the seats in parliament and would not run a presidential candidate. It includes ignoring massacres against the Coptic Christian community; the hasty construction of the constitution; the dismissal of judges; the quick-and-dirty ‘referendum’ that claims 63 % of the vote without noting that less than 25 % of Egyptians voted; and the December protests. It requires, then, allowing Morsi to run roughshod over the Egyptian people, much as his predecessor did.”^58^

By March 2013, even the Center for American Progress was gently criticizing the Obama administration for not not using its leverage and enunciating democratic principles with more consistency. Brian Katulis, Ken Sofer and Peter Juul, pointed to the “looming political legitimacy crisis” in Egypt and said “it remains uncertain how much leverage the United States has managed to build for itself inside Egypt.” Noting that the current policy approach “with its focus on the links between security and economics, has served some US national security interests in the short term,” they said: “Nevertheless this policy approach has limitations, given the messy political transition process and the growing political and social divisions inside Egypt. The Obama administration has done an excellent job advancing America’s security interests in the region, but it can do more to make clear to Egypt’s new leaders that support of democratic values by the current ruling authorities in Egypt is just as vital as maintaining security cooperation. …. As Egypt heads into the next phase of its transition, the United States should continue to outline more clearly and consistently that it seeks to support a truly free and democratic transition in Egypt. It should also underscore that US interests and values are at stake.”^59^

Even critics of American “unilateralism” and of what they saw as Bush’s overly zealous Mideast policies were starting to criticize America’s seeming indifference to the Egyptian peoples’ plight and its unwillingness to pressure Morsi’s government. Michael Wahid Hanna noted that “unconditional support of nominal allies will endanger the very stability that the United States prizes.” His prescription for better policy centered around “conditional engagement” merits attention:

The United States must make clear to regimes that its support cannot substitute for the support of a country’s own citizens, and that the judgments of those citizens regarding their regime’s legitimacy must ultimately dictate the position of the United States. This is a critical message for America’s undemocratic allies in the region, and this conditional engagement represents the only plausible path forward for the United States. The uneven performance of the region’s democratically elected Islamist leaders also suggests a policy approach toward states that have suppressed the forces for change—namely, encouragement of bottom-up democratization. Doing this would include taking steps such as pressing for municipal and provincial elections as a precursor to broader reforms. In pushing such a course on countries that have avoided regime change, the United States can explore anew the feasibility of more gradual reform, which has often been employed rhetorically by authoritarians to avoid actual reform. Further, an approach that seeks to impart governing responsibilities upon opposition groups will ease their potential transition to national leadership. The United States also should not make assumptions about the inevitable role of Islamists. While they remain the most organized and potent political force in many countries in the region, the United States shouldn’t view the Arab world with an essentialist lens that sees in Islamist rule the natural equilibrium. Such an approach will alienate non-Islamist political forces and encourage the monopolization of power by Islamist groups. … Assuming Islamist predominance will also create a misplaced permissiveness with respect to religiously based repression. What might be termed the soft bigotry of Orientalist expectations would undermine notions of universal values and encourage an inherently unstable model of governance that will ill serve U.S. regional interests and undermine the prospects for peaceful and sustainable change.^60^

Nowhere has the Obama administration’s neglect of human rights and democracy in the formation of foreign policy been more devastating than in the Middle East, where it has led to indifference to human longing and human suffering and to the enabling of some of the world’s worst regimes. Obama and Clinton said nary a word in support of Iranian dissidents languishing in notorious Iranian prisons. Unlike their predecessors, they did nothing behind the scenes to encourage Egyptian ally Mubarak to embrace democracy and did very little to encourage his Islamist successors toward toleration of Christians and moderates. They came very close to ignoring the bloodbath in Syria and did ignore it at first. They were mute and passive in response to Assad’s massacre of peaceful protestors, who were part of what was originally a genuinely pro-democracy movement, and to Iran’s brutal snuffing out of the Green movement. They said nothing about Syria’s brutal occupation of Lebanon.

This is perhaps the most troubling flaw of this administration’s foreign policy. It eschews morality and principles and focuses on prosperity, security and “shared interests”-even with dictatorships-instead. There is thus an untold irony in this administration’s foreign policy: *Its supposedly new policy of outreach is based on outreach to those in power, however oppressive they may be, and does not include outreach to the people themselves.* In the guise of a more open, progressive foreign policy it actually prioritizes stability and reinforces those in power, including tyrants. It hoped that if it ignored the Morsi regime’s repression, as the US often did for Mubarak’s regime, it would gain Morsi’s cooperation. It forgets the oft-observed connection between regime type and regime behavior.

By spring of 2013, the situation in Egypt had deteriorated, but there was still room for the United States to try to make a positive difference. In what Amir Taheri cautiously termed a hopeful sign, President Morsi announced fresh parliamentary elections to take place in April. He delayed the timetable of the elections by a week to meet the demands of Christian Copts that the elections not coincide with Coptic Easter. Taheri noted that Morsi had refused to issue an outright ban on political parties or to actually *close* those opposition newspapers he had pressured.^61^ In perhaps another hopeful sign, The Freedom and Justice Party denounced violence that took place outside the Coptic cathedral in Abbaseya, and stressed its support for initiatives “to bridge gaps and preserve the nation.”

While these were perhaps hopeful signs, there was reason to view the Freedom and Justice Party’s denunciation of violence with skepticism. In *Persecuted*, Marshall, Gilbert and Shea disclose the “underreported fact” that “Christians are the single most widely persecuted religious group in the world today”–a fact confirmed by the Pew Research Center, the *Economist*, the Vatican and other sources.^62^ Gilbert asserted in a April 2013 blog on *Ricochet*, “Attacks on Egypt’s Copts … have increased dramatically since the ouster of strongman Hosni Mubarak. The present Muslim Brotherhood regime makes virtually no effort to protect the increasingly vulnerable Christian community. … He [Samuel Tadros] writes, ‘In the past two years from April 2011 until today, 59 Copts have been murdered: 28 in Maspero, 4 in Abu Qurqas, 6 in Imbaba, 12 in Mansheyet Nasser, 1 in Libya, 1 in Dahshour, and 7 in Khosous. …714 Copts have been wounded and no one has been tried for those attacks.”^63^

While stipulating that our influence would be “along the margins,” and that Egypt will determine its political future, Ross and Jeffreys urged the use of US leverage to convince Egypt to maintain international obligations; to fight terror and not provide a safe haven for terrorists; to respect minority and women’s right; and permit political pluralism. To their list, I would have added religious freedom.

They emphasized the difficulties of dealing with the Muslim Brotherhood whose “values and beliefs fundamentally challenge our own.” On the other hand, they stressed: “Egypt’s profound economic needs provide us both the opportunity to be helpful and the leverage to provide ground rules for our assistance. We should be very clear about what matters to us and be prepared to use our assets, and the considerable resources we can mobilize from others, to foster the achievement of those objectives.”^64^

There was no reason to think that the Morsi government’s commitment to democracy was more than half-hearted, or that recent conciliatory gestures would have occurred were the government not under extreme internal pressure. On the other hand, for that very reason, there *was* reason for the United States, finally, to make its support of the Morsi government *conditional*. With enough internal *and* external pressure, that included the imposition of clear, unwavering conditions for US aid, and that included the clear, unwavering pronouncement of democratic principles and strategic priorities, the situation might possibly have improved. If it had improved enough that Christians and secularists and women and Muslim moderates actually believed enough in the legitimacy of democratic processes that they participated in them–and believed their voices would be heard and their votes would be counted–Egypt might have turned a tenuous corner toward a better future without revolution.

But the United States never took a stand for liberty and human rights and never made a move to pressure the Morsi government toward real reform. In a July 2013 article, Glenn Kessler of the *Washington Post* aptly observed that the Morsi government “didn’t have to pay any apparent price for its anti-democratic actions.” Among such actions, Kessler noted, “Morsi launched more prosecutions against reporters and authors for the crime of ‘insulting the president’ than Mubarak, Anwar Sadat, Gamal Abdel Nasser and previous rulers put together.” … Kessler added, “Yet, in Tanzania, Obama said, “decisions [on aid] are based on whether or not a government is listening to the opposition [and] maintaining a free press.”^65^

## Revolution Again

On June 30th, 2013, millions of Egyptians emerged in solidarity onto the streets to protest President Morsi and his regime. Fed up with the regime’s disregard of human rights and constitutional freedoms, disastrous handling of the economy, and failure to address the people’s concerns, they shouted, “Leave, leave!” Many of the protestors carried signs with such slogans as “Obama Supports Morsi.” Journalist and scholar Walid Phares explained in an interview,” We need to understand what Egyptians are trying to say. Most Egyptians are–with the exception of the Brotherhood obviously–very angry, very frustrated. …Not with the American public; they love the American people and citizens, but with the Obama administration because it openly was supporting the Muslim Brotherhood regime.”^66^

In the wake of massive protests, Egypt’s army intervened in support of Egyptians rebelling against Islamist authoritarianism. When it suited its purposes, the military had opportunistically allowed and abetted violence against the very secularists, women and Christians with whom it now claimed an alliance. Now, the military sought the endorsement of religious leaders, political leaders and youth activists, many of whom shared the stage when General El-Sissi announced Morsi’s ouster. The next day, the military announced that the Chief Justice of the Supreme Constitutional Court had been sworn in as president. As James Jay Carano and James Phillips of the Heritage Foundation put it, “Egypt’s secular and liberal opposition [turned to] Egypt’s army in despair, angry that the Obama Administration uncritically supported the Morsi regime.”^67^

Not surprisingly, after Morsi’s ouster, violence and chaos erupted across Egypt as Islamists protested the “coup” against their “elected president” while those who threw him out insisted that Morsi’s presidency was illegitimate and violated every principle of elective government.

As Brotherhood protests grew in intensity and violence, and the military response grew in response, the Obama administration seemed to find the voice for democracy it had previously lost. Seemingly incapable of forthright speech or action for liberty and human rights when the Muslim Brotherhood was in power, the administration spoke out with conviction against the actions of the military: As *Reuters* put it, “Secretary of State John Kerry was unusually forthright in condemning the state of emergency …. ‘In the past week, at every occasion .. we and others have urged the government to respect the rights of free assembly and free expression, and we have also urged all parties to resolve this impasse peacefully and underscored that demonstrators should avoid violence and incitement,” Kerry said. Some of the toughest U.S. messages were delivered personally to Sisi in almost daily telephone calls by Defense Secretary Chuck Hagel, diplomats said. …The United States took the rare step of signaling its displeasure to a strategic Middle East ally, which has a peace treaty with Israel, by halting delivery of four F-16 aircraft under its military aid program last month.”^68^

Having been silent regarding the Morsi government’s abuses, Obama made a rare (for him) statement in defense of political liberty. He took a moral stand, which he had been unwilling to do before. He declared that the new government was embarking on a “dangerous path taken through arbitrary arrests, a broad crackdown on Mr. Morsi’s associations and supporter, and now tragically the violence that’s taken the lives of hundreds of people and wounded thousands more.” Without a word regarding the Morsi supporters’ violent attacks on Christians and secularists, Obama said, “We deplore the violence against civilians. We support universal rights essential to dignity, including the right to peaceful protest. We oppose the pursuit of martial law, which denies those rights to citizens under the principle that security trumps individual freedom, or that might makes right. And today the United States extends its condolences to the families who were killed and those who were wounded.”

Oh, what Egyptian secularists, Christians, women and Muslim moderates would have done for such displays of support from the American president. Oh, what peaceful Iranian protestors and peaceful Syrian protestors would have done for such words in their behalf. But this presidency has been defined by *not* issuing such words, by moral neutrality, and by “engagement” with the world’s worst tyrants.

Obama would have been right to insist that the new government make a distinction between Islamist extremists and peaceful Muslim protestors and that it cease its excessive and often discriminatory use of force. But Obama himself overlooked the distinction, instead painting the Muslim Brotherhood with broad, mostly flattering strokes. He failed, for example, to object to the September 2013 campaign of Brotherhood supporters to force the roughly 15,000 Christian Copts of Daiga to pay a jizya tax, as tribute to Muslims. So too, he failed to reach out to the large numbers of Egyptians who, even though glad Morsi is gone, favor neither the Brotherhood nor the military and could benefit from guidance as they work for the creation of truly accountable and representative government. In a recent article for the *Daily Beast*, Josh Rogin and Eli Lake observed that the Obama administration is resorting to “revisionist history,” now claiming that its decisions toward Egypt were always based on advocacy of the rule of law, civil liberties and democracy.^69^ That, we know, is patently *not true*. Rogin and Lake point out, for example, that, in March, five senators proposed changing the way the US gives aid to Egypt by placing more emphasis on safeguards of democracy, human rights and the rule of law. But the Obama administration, led by Secretary Kerry and Ambassador Patterson, fought those changes. Not only that, Kerry delivered an additional 190 million of aid to Egypt that very month, conditioning it only on economic (not political) reform. Once the Morsi government was ousted, however, the administration announced (against the advice of foreign policy experts) that it was suspending most of the military and financial aid it gives to the military, citing "decisions inconsistent with democracy." As America recedes, Russia steps into the void; Russian and Egyptian officials have begun discussions on defense cooperation, with Russian Foreign Minister Sergei Lavrov and Defense Minister Sergei Shoigu meeting with Egyptian Foreign Minister Nabil Fahmy and Defense Minister Abdel Fattah el-Sisi in the highest level Russian visit in years. 

The US government must work feverishly to restore its badly damaged reputation with the Egyptian people, and to reestablish strategic and geopolitical leverage in the region, for the stakes are terribly high. Now that it has rediscovered words like *freedom* and *rule of law*, it must show that it genuinely promotes and believes in such things. It must lend its considerable resources and knowledge to those seeking to establish civil society in Egypt. Yes, the administration should pressure the Egyptian army to live up to its promises, but it should apply similar pressure to Islamists. Our alliance with Egypt is indispensable. Our demonstration of *genuine* support for a freer political and economic system and greater opportunity for the Egyptian people should be our upmost priority --- not just because it benefits us to have a stable and free ally in the Middle East, but because it is within our best foreign policy traditions. It is the right and the wise thing to do.

## Footnotes

1 Robert J. Lieber, *The American Era: Power And Strategy For The 21st Century.* New York: Cambridge University Press, 2005, p.181.

2 United Nations Development Fund for Economic and Social Development, “Arab Human Development Report 2002: Creating Opportunities for Future Generations,” *United Nations Publication 2002*, overview-2.

3 Shadi Hamid et al., *The Arab Awakening: America and the Transformation of the Middle East*. Washington D.C.: Brookings Institution Press, 2011, p.13

4 Glenn Kessler, “The Fact Checker,” *The Washington Post*. 10 February 2011, http://voices.washingtonpos.com.

5 Alaa Al Aswany, *On the State of Egypt: What Made The Revolution Inevitable*, trans. Jonathan Wright. New York: Vintage Books, 2011, 8.

6 Shadi Hamid et al., *The Arab Awakening: America and the Transformation of the Middle East*. Washington D.C.: Brookings Institution Press, 2011, p.103

7 Michael Youssef, Ph.D., *Blindsided: The Radical Islamic Conquest*. U.S.: Kobri Books, 2012, Chapter Ten.

8 Edward Wakin, *A Lonely Minority*. New York: W. Morrow & Company, 1963, pp. 4, 30.

9 Samuel Tadros, “The Christian Exodus From Egypt,” *The Wall Street Journal,* 11 October 2012, http://online.wsj.com.

10 Paul Marshall, and Lela Gilbert, and Nina Shea, *Persecuted: The Global Assault On Christians*. Nashville: Thomas Nelson, 2013, p.192.

11 Australian Government Refugee Review Tribunal, *Country Advice Egypt,* 26 February 2010.

12 Marc Lynch, “Did We Get the Muslim Brotherhood Wrong?” *Foreign Policy*, 10 April 2013. www.foreignpolicy.com.

13 Daniel Kurtzer, “Is Revolution What’s Best For The Rest?” *Foreign Policy*, 4 February 2011. www.foreignpolicy.com.

14 Magdi Khalil, “The Muslim Brotherhood and the Copts.” *ThreatsWatch*, 20 April 2006. http://threatswatch.org.

15 Ibid.

16 “Egyptian Presidential Candidate Doubts Al Qaeda Role in 9/11.” www.washingtontimes.com, May, 2012.

17 Sarah Lynch, and Oren Dorell, “Egyptian president’s aims unknown.” *USA Today*, 27 June 2012. http://usatoday30.usatoday.com.

18 Vivian Ibrahim,“Egypt: One Year After Mubarak.” *Fair Observer*, 10 February 2012. http://www.fairobserver.com.

19 James L. Gelvin, *The Arab Uprisings: What Everyone Needs To Know*. New York: Oxford University Press, 2012, 148.

20 Shadi Hamid et al., *The Arab Awakening*, 77.

21 Stratfor Intelligence. www.stratfor.com

22 Liane Hansen, “Clinton Sticks to U.S. Principles on Egyptian Reform.” *NPR News/Weekend Edition*, 6 February 2011. http://www.npr.org/.

23 Arshad Mohammed, “U.S. Shifts to Closer Contact with Egypt Islamists.” *Reuters*, 30 June 2011. http://www.reuters.com.

24 Josh Gerstein, “DNI Clapper retreats from ‘secular’ claim on Muslim Brotherhood.” *Politico*, 10 February 2011. http://www.politico.com.

25 Nina Shea, “Egypt’s Copts: Will the Regions Largest Non-Muslim Religious Community Simply Disappear?” *New Republic*, 10 June 2011. http://www.newrepublic.com

26 Frida Ghitis, “World Citizen: U.S., Arab Liberals Losing Ground in Middle East.” *World Politics Review*, 4 August 2011. http://www.worldpoliticsreview.com.

27 Arnaud de Borchgrave, “Egypt on Slippery Slope Toward Civil War.” *Newsmax*, 11 October 2011. http://www.newsmax.com.

28 “Barack Obama and William Hague on The Maspero Massacre of the Copts by Egyptian Army 9 10 11–What their Pathetic Response Tell Us? What Should We Do?” *Coptic Literature Wordpress* 16 October 2011. http://copticliterature.wordpress.com.

29 Jay Carney, “Statement by the Press Secretary on Violence in Egypt,” Washington D.C., (The White House: Office of the Press Secretary 10 October 2011).

30 “Muslim Brotherhood vows not to recognize Israel.” *The Jerusaluem Post*, 01 January 2012. http://www.jpost.com.

31 Patrick Goodenough, “WH: ‘Democratic Process’ More Important Than Islamist Victories in Arab Nations,” *CNS News*, 29 November 2011. http://cnsnews.com.

32 Avi Asher-Schapiro, “Egypt’s Copts Face an Uncertain Political Future.” *Muftah*, 21 May 2012. http://muftah.org.

33 Ryan Mauro, “Obama Administration Training Egyptian Islamists for Elections.” *FrontPage Magazine*, 18 November 2011. http://frontpagemag.com.

34 Stephen Brown, “The Obama-Brotherhood Love Affair Heats Up.” *FrontPage Magazine*, 13 January 2012. http://frontpagemag.com.

35 Peter Baker, and David D. Kirkpatrick, “Egyptian President and Obama Forge Link in Gaza Deal.” *The New York Times*, 21 November 2012. http://nytimes.com.

36 “Egypt must urgently cancel ‘dangerous’ new powers given by military,” *Amnesty International*, 14 June 2012. http://www.amnesty.org.

37 Carolyn Glick, “America and the Arab Spring.” *Real Clear Politics* 25 January 2012. http://realclearpolitics.com.

38 Richard Spencer, “Egypt’s Muslim Brotherhood refuses to boycott presidential election.” *Telegraph*, 15 June 2012. http://www.telegraph.co.uk.

39 Sarah El Deeb, and Hamza Hendowi, “Egyptian facing a tough choice in picking a President.” *Associated Press*, 17 June 2012. http://www.post-gazette.com.

40 Borzou Daragahi, “Egyptian President-elect Mohamed Morsi’s full 25-minute speech tonight.” *Facebook*, 24 June 2012. http://www.facebook.com/notes/borzou-daragahi/.

41 Shadi Hamid, “An Unfinished Revolution.” *Georgetown Journal of International Affairs* Summer/Fall 2011.

42 Maggie Michael, AP “New Egyptian president already under criticism.” *The Daily Gazette* 29 July 2012. http://www.dailygazette.net.

43 Neil Hicks, “Egypt’s Transition to Democracy One Year On: Recommendations for U.S. Policy.” *Human Rights First* 26 January 2012. http://www.humanrightsfirst.org.

44 Niall Ferguson, *Real Clear Politics Video*, 14 February 2011, www.realclearpolitics.com.

45 Josh Rogin, “Clinton Waives restrictions on U.S. aid to Egypt.” *The Cable* 22 March 2012. http://thecable.foreignpolicy.com.

46 Ibid.

47 Ibid.

48 Michael Wahid Hanna, “Clouded U.S. policy on Egypt.” *Foreign Policy* 26 February 2013. http://mideast.foreignpolicy.com.

49 Secretary of State Hillary R. Clinton, “Remarks at the Release of the 13th Annual Report on International Religious Freedom,” Washington D.C., (U.S. Department of State, September 13, 2011).

50 Michael Meunier, “Obama Gives Cold Shoulder to Egyptian Secular Democrats.” *Investigative Project, IPT News* 21 December 2012.

51 Mary Abdelmassih, “100,000 Christians Have Left Egypt Since March: Report.” *Assyrian International News Agency* 27 September 2011. http://www.aina.org/news/20110926194822.htm.

52 Secretary of State Hillary R. Clinton, “Remarks at the Release of the 2011 International Religious Freedom Report,” Washington D.C. (Carnegie Endowment for International Peace, U.S. Department of State, July 30, 2012).

53 Dennis B. Ross, and James F. Jeffrey. “Obama II And The Middle East: Strategic Objectives For U.S. Policy.” Strategic Report 12, Washington Institute for Near East Policy, Washington D.C. March 2013, 23.

54 David D. Kirkpatrick, “More Morsi Aides Resign as Egypt Deploys Tanks in Cairo.” *The New York Times* 6 December 2012. http://www.nytimes.com.

55 “Why We Shall Boycott The Referendum On The Islamist Constitution In Egypt,” *Coptic Literature Wordpress* 12 December 2012. http://copticliterature.wordpress.com.

56 Ahmed Eleiba, “F-16 deal redefines US relationship with Egypt’s Morsi administration.” *Ahram Online* 14 January 2013. http://english.ahram.org.eg.

57 Abigail Hauslohner, “Egypt’s Morsi remakes cabinet, increasing Islamist presence.” *The Washington Post* 6 January 2013. http://www.washingtonpost.com.

58 Shashana Bryen, “Enshrining Ideologies: Egypt and the US.” *Gatestone Institute* 22 January 2013. http://www.gatestoneinstitute.org.

59 Brian Katulis, Ken Sofer, and Peter Juul, “Preparing U.S. Policy for the Next Phase of Egypt’s Transition.” *Center for American Progress* 01 March 2013. http://www.americanprogress.org.

60 Michael Wahid Hanna, “The Seven Pillars of the Arab Future: The United States cannot make a success of the Arab Spring. Only region’s nations can. Here are the ways they need to mature.” Spring 2013. democracyjournal.org. 76–78.

61 Amir Taheri, “Will Egypt’s democrats get serious?” *New York Post* 27 February 2013. http://www.nypost.com.

62 Marshall, Gilbert and Shea, *Persecuted*, 4.

63 Lela Gilbert, “Killed Because They Were Copts.” *Ricochet* 07 April 2013. http://www.Ricochet.com.

64 Ross and Jeffrey, “Obama II And The Middle East: Strategic Objectives For U.S. Policy,” 21&22.

65 Glenn Kessler, “Obama’s Claim that Aid to Egypt Was Based on Adherence to ‘Democratic Procedures.” *The Washington Post* 4 July 20 2013. www.washingtonpost.com

66 Bill Hoffmann, “Walid Phares: Egyptians Mad at US Embrace of Muslim Brotherhood.” 14 August 2013. www.newsmax.com

67 James Jay Carafano, Ph.D. and James Phillips, “Egypt: A Way Forward After a Step Back.” 9 July 2013. www.heritage.org.

68 Paul Taylor, “Exclusive–West Warned Egypt’s Sisi to the End: Don’t Do It.” *Reuters* 14 August 2013. www.reuters.com

69 Josh Rogin and Eli Lake, “Obama Offers a Revisionist History of His Administration’s Approach to Egypt.” *The Daily Beast* 2 July 2013. www.thedailybeast.com.

